# Talks with Dr. Mandeville

**Published:** 1892-03

**Authors:** 


### TALKS WITH DR. MANDEVILLE.

### No. 2.

Q. Doctor, do you think it is injurious to eat hearty food immediately before retiring?

A. Popularly, it is thought injurious, but unless dinner or supper has been late, or the stomach disordered, it is not only harmless, but beneficial if one is hungry. Invalids and persons in delicate health, should always eat at bed time. This seems heretical, but it is not so. Animals after eating, instinctively sleep. Human beings become drowsy after a full meal. Why? Because blood is solicited toward the stomach to supply the juices needed in digestion. Hence the brain receives less blood than during fasting, becomes pale, and the powers grow dormant. Sleep, therefore ensues. This is a physiological truth. The sinking sensation in sleeplessness is a call for food. Wakefulness often is merely a sympton of hunger. Gratify the desire and you fall asleep. The feeble will be stronger at dawn if they eat on going to bed. Fourteen hours lie between supper and breakfast. By that time the fuel of the body has become expended. Consequently the morning toilet fatigues many. Let such eat at bed-time and take a glass of warm milk or beef tea before rising. Increased vigor will result.

"But the stomach must rest."

True, yet when hungry, we must eat. Does the infant's stomach rest as long as the adult's? The latter eats less often merely because his food requires more time for digestion. Seldom can one remain awake until 10.30 or 11, without hunger. Satisfy it and sleep will be sounder.

Q. Do the stomach and intestines continue their functions during sleep?

A. Yes, the stomach and intestines continue their functions during sleep, though with lessened activity; the secretions are not suspended, all the essential functions continue to be exercised; while there is a diminished activity of the secreting glands, yet in healthful persons these organs are still adequate to their work, as shown by the fact that many persons can eat a full meal on going to bed, sleep soundly, and be ready for another meal on waking.

During the night give wakeful children food, and sleep will follow. The sick should invariably eat during the night. This is imperative. Delicate persons and children may sip slowly, warm milk, beef tea or gruel. Vigorous adults may also eat bread and milk, cold beef, mutton, chicken and bread, raw oysters, all of course, in moderation, but eat only when hungry. The appetite should be the monitor. It is nature's call for food and should not be neglected.

